# A Comparative View on the Oviductal Environment during the Periconception Period

**DOI:** 10.3390/biom10121690

**Published:** 2020-12-17

**Authors:** Leopoldo González-Brusi, Blanca Algarra, Carla Moros-Nicolás, Mª José Izquierdo-Rico, Manuel Avilés, Maria Jiménez-Movilla

**Affiliations:** Department of Cell Biology and Histology, School of Medicine, University of Murcia, Campus Mare Nostrum and IMIB-Arrixaca, 30100 Murcia, Spain; leopoldo.gonzalez@um.es (L.G.-B.); b.algarraonate@um.es (B.A.); carla.moros@um.es (C.M.-N.); mjoseir@um.es (M.J.I.-R.)

**Keywords:** oviduct, embryo, oviductal fluid, OVGP1

## Abstract

The oviduct plays important roles in reproductive events: sperm reservoir formation, final gamete maturation, fertilization and early embryo development. It is well known that the oviductal environment affects gametes and embryos and, ultimately, the health of offspring, so that in vivo embryos are better in terms of morphology, cryotolerance, pregnancy rates or epigenetic profile than those obtained in vitro. The deciphering of embryo–maternal interaction in the oviduct may provide a better understanding of the embryo needs during the periconception period to improve reproductive efficiency. Here, we perform a comparative analysis among species of oviductal gene expression related to embryonic development during its journey through the oviduct, as described to date. Cross-talk communication between the oviduct environment and embryo will be studied by analyses of the secreted or exosomal proteins of the oviduct and the presence of receptors in the membrane of the embryo blastomeres. Finally, we review the data that are available to date on the expression and characterization of the most abundant protein in the oviduct, oviductin (OVGP1), highlighting its fundamental role in fertilization and embryonic development.

## 1. Introduction

The oviduct, a tubular organ that connects the ovaries with the uterus, is composed of four anatomical regions: the infundibulum, the ampulla, the isthmus and the uterine-tubal junction [1]. In female mammals, this passageway is also known as the uterine tube or Fallopian tube. While the oviduct was previously considered a passive channel for the transport of gametes and embryos, it is now widely accepted that it is not just a tract that joins the ovaries to the uterus. Indeed, it plays important roles in reproductive events participating in sperm reservoir formation, final gamete maturation and transport, fertilization and early embryo development; moreover, inside this organ is where the first maternal-embryo cross-talk begins [2,3,4,5,6,7,8,9]. Before implantation, the embryo spends between 1 and 10 days in the oviduct, depending on the species (Table 1). During these days important morphological, molecular and metabolic modifications occur, such as, the first mitotic division and embryonic genome activation [10,11,12,13,14]. In this phase, the conceptus is dependent on oviductal fluid (OF) formed by the nutrients secreted from the oviduct epithelial cells, as well as serum transudate [15,16,17]. The OF is a complex and a dynamic fluid, composed of metabolites, inorganic salts, amino acids, proteins, glycosaminoglycans, lipids and extracellular vesicles, amongst others, which fulfil the preimplantation microenvironment, guaranteeing a conceptus viable for implantation and leading to the creation of healthy offspring [3,7,18,19,20]. Despite the important functions in which it participates, the oviduct has not been the object of a detailed study until very recently, perhaps due to the success of in vitro fertilization (IVF) and other assisted reproductive technologies (ARTs), which bypass the oviduct to produce mammalian embryos in vitro. However, it is well known that the oviductal environment affects gametes and embryos and, ultimately, the offspring health. In fact, in vivo-produced embryos are better in terms of morphology, cryotolerance, pregnancy rates or epigenetic profile than those obtained in vitro [21,22,23,24]. The low efficiency of in vitro embryo production has been widely reported in several species; for instance, in porcine the success rate does not exceed 45% [25], polyspermy and insufficient oocyte cytoplasmic maturation being the main causes. In bovine, the ratio of embryos that reach the transferable stage is around 30% to 40% [26], and, in equine species the blastocyst rate reached by intracytoplasmic sperm injection (ICSI) is also around 40% [27].

In an attempt to improve ARTs the tendency followed by researchers has been to mimic the maternal oviductal environment, culturing the embryos with oviductal epithelial cells [62,63] or by supplementing IVF (for a review see [64]) or in vitro culture (IVC) media with different molecules, EVs or natural fluids. EVs play an important role in cell-to-cell communication [65] by transferring their molecular load from one cell to another [7,66]. In the reproductive field, EVs secreted by the oviduct (oEVs) and embryos (eEVs), are key players in the crucial two-way dialogue between the oviduct and the embryo (for a review see [67]). In porcine, the addition of oEVs to the IVF medium regulates polyspermy [68], while oEVs isolated from the conditioned medium of bovine oviductal epithelial cells [69] or from in vivo-derived OF [7,70] improve bovine embryo quality during IVC, in terms of blastocyst rates, cell number, hatching rates, embryo cryosurvival and gene expression. On the other hand, in pig, the efficiency of IVF has been reported to be higher with the addition of OF [68]. The beneficial effect of OF on bovine embryo development and quality has also been reported in terms of cryotolerance, number of trophectoderm cells, gene expression [71] and DNA methylation [72]. A comparison in pig of in vitro-produced embryos with/without reproductive fluids pointed to a higher quality in terms of cell number and ability to hatch in the ART-derived blastocysts when reproductive fluids were added to the culture medium [24]. In this same work, the authors compared the transcriptome and epigenetic profile of in vitro-produced blastocysts without reproductive fluids (C-IVF) and with reproductive fluids (Natur-IVF) and their in vivo counterparts, finding that, the transcriptomic profile showed greater variability in the C-IVF group. Furthermore, the number of differentially expressed genes was higher between the C-IVF and in vivo, than between Natur-IVF and in vivo. Moreover, C-IVF embryos showed more aberrantly expressed genes, which related with epigenetic reprogramming, better embryo development, cell growth and imprinting, and higher methylation levels. All these studies indicate that, an environment similar to physiological conditions during IVC maximizes embryo development and quality. However, many of the components of the OF remain unknown. In the same way, little information is available about the complex molecular dialogue that occurs between the oviduct and the preimplantated embryo (reviewed in [31,73]. Such knowledge is crucial to better understand embryo development and improvement of ARTs.

The aim of this review was to take a deep look at recent discoveries about the oviduct in different species in order to better understand the importance of this organ in the periconception period. With this in mind, a comparative view of the impact of the embryo on oviductal gene expression and the impact of the oviduct on the embryo are analyzed and discussed, taking into consideration the most recent publications in the field. Finally, a comparative study among mammalian species was made of the molecular structure, synthesis and role of oviductin (OVGP1), the most abundant protein in the OF during fertilization and early embryonic development. 

## 2. Materials and Methods

Transcriptomics data concerning the oviduct were taken from the studies done in bovine [74], equine [75] and porcine species [76]. These works were chosen because they use oviducts in vivo and they compare directly the transcriptomic profile of an oviduct with embryos vs. an oviduct in the equivalent stage of the oestrous cycle. Differentially expressed genes (DEGs) from each table were reanalyzed according to the most recent annotation in order to identify former LOC uncharacterized genes (named LOC) whose orthologs have been discovered. Moreover, in the case of the equine species, plenty of mRNAs coding for equine transposases and species-specific endogenous retroviruses were discarded for the cross-species comparison. To compare the DEGs across species, Venn diagrams with all the DEGs and with the down-regulated and up-regulated genes were made.

RNAseq data of in vitro cultured embryos were extracted from the GEO DataSets repository as follows: bovine data from GSE52415 [77], porcine data from GSE139512 [78] and human data from Xue et al., 2013 [79]. Briefly, the fastq files for each dataset were downloaded and transcript expression was quantified with kallisto [80], gene expression was later summarized with an in-home script written in R which makes use of the biomaRt package [81] in order to identify which transcripts belong to a common gene. This approach, using the same workflow for all the datasets, was done in order to minimize differences due to different processing of the data by the original authors. A mean expression in all the available replicates over a detection threshold of 1 TPM (transcript per million) was established in order to filter out non-expressed genes. 

Proteomics data involving oviductal fluid samples were obtained from the supplementary files of the studies done in bovine [82,83], equine [84], human [85] and porcine. The study by Canha-Gouveia and coworkers (2019) [85] was to our knowledge the more complete and recent compilation of OF proteins in the human species, including samples corresponding to the secretory phase of the menstrual cycle. When working with bovine data we chose the most recent studies on whole OF by Lamy et al. (2016) [82] and Pillai et al. (2017) [83]. We did not consider discriminating between estrous cycle phases as in the supplementary materials, the data was merged. For the porcine species, we compiled our data from several proteomic assays done with porcine OF from the early luteal stage. We provide a Table S1 with the detected peptide and proteins from each sample. The detailed methods concerning the HPLC Liquid Chromatography-Mass Spectrometry (LC-MS/MS) workflow are described by Luongo et al., 2020 [86].

The database of ligand-receptor interactions in humans compiled by Ramilowski et al., 2015 [87] was used to infer interactions between embryo receptors and oviductal proteins. Selected interactions for each species involved a ligand-receptor pair as described: a ligand present in the oviductal fluid proteome interacting with a receptor present in the blastomere according to the RNAseq analysis.

## 3. Impact of Embryo and Oviduct Communication

### 3.1. A Comparative View of the Impact of the Embryo on Oviductal Gene Expression

**Figure 1:**
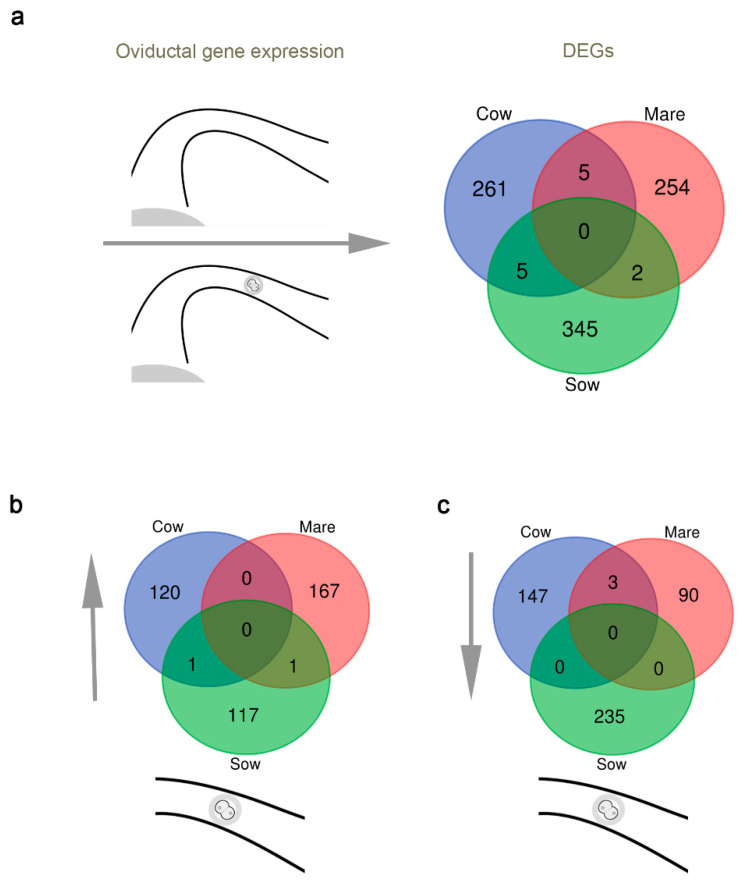
DEGs between oviducts containing embryos and oviducts on the equivalent day post-ovulation across in bovine, equine and porcine species. (**a**) Total DEGs. (**b**) Up-regulated genes in the presence of embryos. (**c**) Down-regulated genes in the presence of embryos.

It was previously reported that a change in the oviductal gene expression pattern occurs in the presence of the embryo in cow, sow and mare [74,75,76], demonstrating the communication between the embryo (or embryos) and the oviduct. We have performed an in silico analysis based on the above mentioned transcriptomic studies in the sow, cow and mare, comparing the gene expression in the oviduct in the presence of an 8-cell embryo (4-cell embryo in the sow) vs. the absence of fertilization. It was observed that more than 250 genes are differentially expressed (DEG) in both conditions in the three species (Figure 1a, Table S2). However, was a surprise to find that very few genes coincided among the three species and only one gene, which corresponds to the MHC-I, is shared among them. When the DEGs were analyzed in more detail, up regulation was observed in 120, 167 and 117 genes in cow, mare and sow, respectively (Figure 1b). In the case of down regulated genes, the data pointed to 147, 90 and 235 in cow, mare and sow, respectively (Figure 1c). These data clearly show that the embryo has an impact on the oviduct, as previously reported in mouse [88], pig [89] and cow [90], but the oviductal response is species-specific. It is not clear if these specific differences are really important but previous studies using heterologous transfer of the embryos showed that the embryos developed well in a foreign oviduct [91]. We are aware of the limitations of our approach: the use of polyovulatory vs. monoovulatory species, different transcriptomics setups (RNAseq vs. microarrays) and different days of sampling after ovulation. Nevertheless, is it also remarkable that all those studies detect a change in genes involved in immune response within the oviduct, suggesting a conserved mechanism of embryo recognition. More research is needed to clarify the impact of these specific genes in each species. Compiling temporal data in those species could be interesting to know more about the evolving scenario across the early embryo development, and to produce the best reproducible results across independent studies.

### 3.2. Impact of the Oviduct on the Embryo

**Figure 2:**
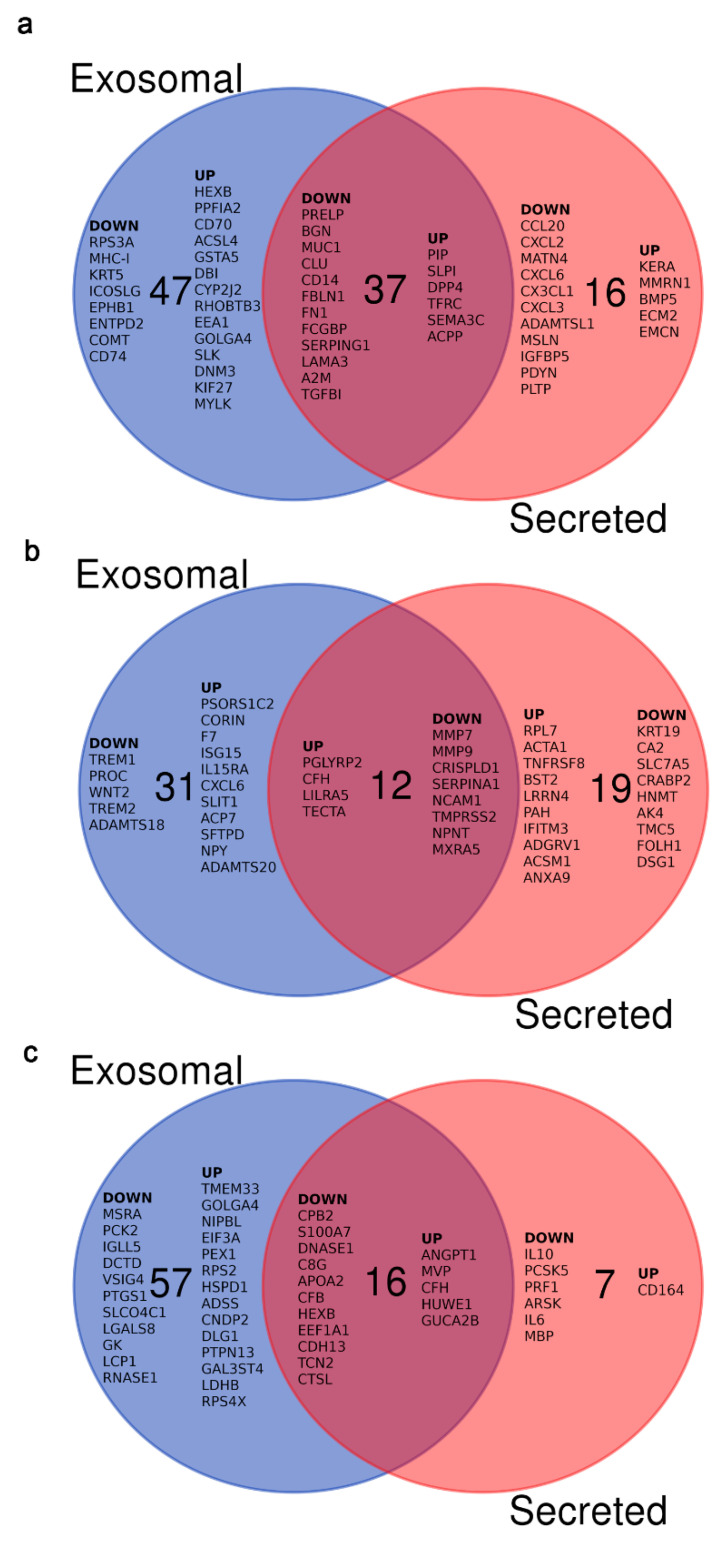
Analysis of the DEG in the oviduct of non-pregnant and pregnant animals related with secreted and/or exosomal proteins. (**a**) Differentially expressed genes coding for exosomal and/or secreted proteins in the ipsilateral oviduct of pregnant vs non-pregnant cows. Data taken from Maillo et al., 2015 [74]. When more than 20 genes were present in an area of the Venn diagram, gene names were selected according the highest fold change. (**b**) Differentially expressed genes coding for exosomal and/or secreted proteins in the ipsilateral oviduct of pregnant vs. non-pregnant mares. Data taken from Smits et al., 2016 [75]. (**c**) Differentially expressed genes coding for exosomal and/or secreted proteins in the pregnant vs. nonpregnant oviduct of sows. Data taken from Martyniak et al., 2018 [76]. When more than 20 genes were present in an area of the Venn diagram, gene names were selected according the highest fold change.

The embryo changes its gene expression pattern in the oviduct or in co-culture with oviductal epithelial cells [92]. A further analysis was performed to obtain information of potential proteins that have a clear impact in the embryo due to their release into the oviductal lumen and their direct contact with the embryo. It is known that proteins can be secreted extracellularly by different mechanisms [93]. A classical mechanism is exocitosis due to the presence of a signal peptide at the N-terminal region of the protein. This peptide directs the protein towards the secretory pathway due to the participation of two relevant organelles, the endoplasmic reticulum and the Golgi apparatus. An alternative way that has been studied recently in several organs involves the release of the proteins and other components through EVs, including exosomes [94]. It is known that the zona pellucida (ZP) is a porous extracellular matrix that surrounds the oocyte and the early embryos, and is permeable to proteins and also to viruses [95]. Thus, it was reported that one of the most abundant secreted proteins, OVGP1 crosses the ZP, enters the perivitelline space and is endocytosed by the blastomeres [96]. Recently, it was found that exosomes are able to pass through the ZP to reach the embryo [7]. Consequently, both types of secretion have a potential impact on the embryo. For this reason, expressed genes or proteins were investigated using the bioinformatic tool DAVID (v 6.8, [97]) considering “secreted” or “exosome” (Figure 2). 

In the cow, it was observed that a total of 100 DEGs are classified as secreted or exosomal proteins (Figure 2a): 16 and 47 are classified as secreted and exosomal proteins, respectively, and 37 as being secreted from both sources. In the mare, it was observed that a total of 62 DEGs are classified as secreted or exosomal proteins (Figure 2b): 19 secreted and 31 exosomal proteins, with 12 coming from both sources. 

In the sow, 80 DEGs were classified as secreted or exosomal proteins (Figure 2c): 7 and 57 classified as secreted and exosomal proteins, respectively, and 16 as from both sources. All these proteins had a specific pattern showing up or down regulation (Table S3). However, it is important to take into consideration a technical point that could be responsible for some of the differences, which may be due to the samples analyzed. Thus, the analysis performed in the cow and mare was made using oviductal epithelial cells, while in the sow the whole isthmus region of the oviduct was used.

Additionally, we analyzed the potential receptors on the embryo and the ligands on the oviductal fluid proteins in the cow, pig and human (Table 2). Thus, in the bovine model we detected 99 receptors on the blastomere plasma membrane that may interact with the 58 oviductal proteins (Table S6). 

The OF proteomics data are taken from Canha-Gouveia et al., 2019 [85] for woman, Lamy et al., 2016 [82] and Pillai et al., 2017 [83] for cow. The data for sow are based on unpublished research by our group involving 465 proteins (a more modest number compared to the aforementioned studies in human and bovine which detected more than 1200 proteins in each). RNAseq data for 8-cell embryos were taken from Xue et al., 2013 [79] for human samples, from Graf et al., 2014 [77] for bovine embryos, and Kong et al., 2020 [78] for porcine samples. Protein-ligand interactions were extracted from the database generated by Ramilowski et al., 2015 [87].

In human, we detected interactions between 63 receptors on the blastomere surface and 49 oviductal proteins for a total of 119 possible interactions (Table S5), while in porcine there are 32 receptors on the blastomere surface that may interact with 22 oviductal proteins for a total of 56 potential interactions (Table S7). In addition, 45 interactions were shared between human and cow, 21 were shared between pig and cow and 11 were conserved between the three species (Table S4).

The mRNA codifying the receptors was demonstrated using RNAseq analysis expression data from eight-cell embryos. In this stage of pre-implantational development, the embryos have gone through the main events of embryonic genome activation [77,79,103], so it can be considered that they are responding to their environment, as there has been a radical change in their transcriptomic profile compared with the oocyte. A limitation of our approach is that all the embryos collected were obtained in vitro and not in vivo so the reception expression pattern could differ in the in vivo conditions. 

The interaction between the OF ligands and their receptors in the blastomeres could be more complex than expected. For example, in the case of the fibronectin present in the OF [104], it can interact with at least 19 receptors of the bovine embryo. Information about the specific pair and ligand interaction is lacking, but it has been observed that fibronectin, a protein typical of the extracellular matrix, is present in the bovine OF [83].

Fibronectin has been shown to induce embryo cleavage but only at an appropriate concentration [105]. In their study, Larson et al. [105] also demonstrated the presence of fibronectin receptors on eight-cells embryos using immunocytochemistry. Fibronectin is present in human and bovine OFs, and its RGD motifs (tripeptide Arg-Gly-Asp, amino acid sequence within the extracellular matrix protein) bind to heterodimeric integrins containing Integrin Subunit Alpha V (ITGAV) [106], both present in human and bovine eight-cell embryos. In contrast, no fibronectin has been detected in porcine species, but as a counterpart, porcine OF has greater quantities of osteopontin (which has also been detected by western blot by Gabler and collaborators in 2003 [107] in bovine OF). This is another protein containing RGD motif, which might work in the same way as fibronectin by stimulating the same receptor.

Among the eight common interactions between the three studied species, the best known ligand present in the OF is lactoferrin (or lactotransferrin, LTF), a glycoprotein secreted by the oviduct to the OF in mouse, human and sheep [104,108,109]. Ward and collaborators [102] detected the expression of this protein by pre-implantational murine embryos, and also the uptake of exogenous protein added to the culture medium, although the receptor present in the blastomeres was not characterized. While LTF is not an essential protein for fertilization, as shown by Ward et al., in a knock-out mouse model [110], it could be interesting to supplement it to an in vitro culture medium at physiological concentrations.

In addition, annexin A1 (ANXA1) is a protein which has been recently identified as an embryo-interacting maternal protein from bovine OF by Banliat, and collaborators in 2020 [98], and has also been immunodetected within the perivitelline space and inside.

Complement component 3 (C3) is another protein present in the OF, the concentration of which is under hormonal regulation in the human, mouse and pig [99,111]. Although Lee, and collaborators in 2004 [99] observed a positive effect of C3 in the size and hatching rates of mouse blastocysts, Georgiou and collaborators in 2011 [112] observed a detrimental effect on porcine blastocysts, suggesting that in this species the C3 concentrations are high during oestrus [111] and decrease in response to the oocyte [100].

Another interesting ligand is the blood protein fibrinogen. Fibrinogen alpha and beta were detected within bovine and porcine OF, while fibrinogen gamma, was also detected in human OF. The role of this protein in reproduction in equine species is known to mediate the implantation of the conceptus in the uterus at about day 40 [101]. The equine conceptus produces fibrinogen on its own (a rare feature, because in adult mammals it is only produced by the liver) and this fibrinogen binds integrins within the mare endometrium.

Furthermore, it may seem surprising that most OF proteins in our list of interactions are actually blood proteins that reach the OF by transudation. It was first thought that this would better explain the above-mentioned successful development of pre-implantational embryos after heterologous transfer, as blood plasma proteins would play a more relevant role than the transcriptomic change in oviductal secreted proteins, which seems to differ greatly between equine, bovine and porcine if we only take into account DEG. However, among the proteins secreted by the oviductal epithelium there are also several ligands with an unknown receptor, so that, they are omitted from our list. Perhaps, one of the most abundant and most intensively studied oviductal proteins is OVGP1, which, after traversing the ZP, is endocytosed by the oocytes or the embryos, in a mechanism that requires the presence of a specific region [113] as explained in more detail below.

Nevertheless, oviductal transcriptomics would also affect oviduct permeability, which explains why the transudate varies during the oestrus cycle and pre-implantational development, as observed by changes in OF volume and total protein concentration [114]. Some authors have also observed changes in concentrations in some plasma proteins in the presence of embryos but not in their absence in the bovine and equine oviduct [84,115].

## 4. OVGP1

The oviduct-specific glycoprotein OVGP1, also known as oviductin, is the major non-serum glycoprotein present in OF. The components of OF have been studied for decades, and it was Oliphant et al., 1984 [116] who described a protein which was synthesized de novo by the oviduct in rabbit under oestrogen control and was localized in the apical secretory granules, released to the oviductal lumen. In 1986, Brown and Cheng [117] confirmed that oestrus-induced oviductal glycoproteins interact with the porcine egg ZP, and, in 1988 Oikawa and collaborators [118] described a glycoprotein of oviductal origin which altered biochemical properties of the ZP of hamster eggs. Thenceforth different names have been given to this glycoprotein, and through the years more information became available (See [119]). Although, the generation of the null mutation of the oviduct-specific glycoprotein gene indicates that OVGP1 is not essential for the process of in vivo fertilization in mice [120], numerous studies have demonstrated the fundamental role of OVGP1 in reproductive processes and embryonic development in different species of mammals.

### 4.1. Origin and Localization of OVGP1

OVGP1 is a glycoprotein that has been detected in a number of mammalian species (Table 3), where it is expressed by the non-ciliated epithelial cells of the oviduct. mRNA of OVGP1 has been localized in basal perinuclear compartments and in the apical cytoplasm of fimbria and ampulla epithelial cells in sheep [121], while the protein has been identified in Golgi saccules and in secretory granules of the non-ciliated oviduct cells in hamster [122,123,124] and inside secretory granules in bovine [125,126], porcine [127], baboon [128], human [129], mouse [130] and rabbit [116]. The fact that the protein has not been detected in rat [131] is due to OVGP1 gene is lost/absent in rats. It is consistent with the reported information about OVGP1 gene existing before the divergence of mammals and being from this species [132]. In megabat it is also considered a pseudogene [133].

Traditionally, it has been described that OVGP1 is solely produced by the oviduct [163,177] while the detection of the protein in the lining of the surface epithelia of the uterus and the uterine fluid [104] was attributed to the transfer of components from the OF. The protein was observed in the epithelial cells of the endometrium in mice, but only at the time of embryo implantation [178]. As regards implantation, women suffering recurrent implantation failure showed significant low OVGP1 mRNA in the endometrium [178]. Whatever the case, the oviduct would be the main organ responsible for the synthesis of this glycoprotein as a component of the OF. The oviduct of the pig [164], sheep [179] and cow [143], shows an increased biosynthetic activity at oestrous, suggesting that the production of more abundant glycoprotein is oestrogen-dependent and is at its highest level at the time of fertilization in some species such as human, cow, pig, sheep, baboon and mice [104,131,135,136,138,166,173,175,180,181,182,183,184,185]. At the time of maximum OVGP1 gene transcription in the oviductal tissue, there is an increase in the number of apical secretory granules in the oviductal epithelium containing OVGP1 and also in the amount of OVGP1 in OF [119]. The OVGP1 gene has been reported to be down-regulated in pregnant compared with cyclic heifers [74]. However, the expression of OVGP1 in some species, such as hamster and rabbit is controversial, as some authors describe no differences in mRNA production during the oestrous cycle [186,187]. Others observed that mRNA levels changed significantly between the oestrous and diestrous stages in the ampulla, but not in the isthmus, of a constitutively expressed OVGP1 [188], and also mention stage-specific OVGP1 expression in the secretory granules of the hamster oviductal secretory cells, with a maximum level at oestrus [189]. In addition, the induction of the production of the protein in new born hamsters by oestradiol has been reported [155].

Moreover, there are also located differences in where oviductal secretion take place [115]. The oviductal biosynthetic activity is major in some functional segments like the infundibulum and the ampulla at oestrous in pigs [164,190] so that, the type and distribution of OVGP1 glycoprotein differs between the ampulla and isthmus in pig and sheep [165,179]. Moreover, the levels of OVGP1 expression detected in these regions vary between species. OVGP1 is synthesized and released by the oviduct in a temporally and regionally specific way in the ewe [191]. In rabbit, the ampulla secretes greater amounts of OVGP1 than in the isthmus [192,193]. Similarly, using RNAseq technology, Gonella- Diaza and collaborators [194] showed greater OVGP1 expression in the ampulla than in the isthmus, but no differences were perceived when distinct periovulatory endocrine profiles were analyzed in cattle. On the contrary, the same mRNA levels of OVGP1 were detected in the fimbria, ampulla and isthmus in baboon [182] and no spatial differences between the transcriptome of the isthmus and ampulla in cow was reported [6]. In mouse, cow and sheep, the protein was not localized in the isthmus [126,130,163,174,175], while in cow, sheep, pig and mouse it has been immunolocalized in the infundibulum [119].

The oEVs, recently mentioned as components contained in the OF and described as an important modulator between gametes and embryos and the maternal tract, are released by oviductal epithelial cells at different stages of the oestrous cycle [8]. OVGP1 transcript and protein have been detected inside porcine and bovine oEVs produced in vivo, suggesting that its secretion is, at least in part, by oEVs [7,8,68]. The molecular composition of bovine oviductal oEVs is regulated by steroid hormones [8]. However, no temporal differential mRNA nor OVGP1 protein concentration was detected in oviductal oEVs at any time during the bovine oestrous cycle [8], although this protein was identified as one of the most expressed proteins in the oviduct [8]. MYH9, the OVGP1-protein binding partner in gametes [195] was also detected in oEV samples [7,8,68]. 

### 4.2. Characterization of OVGP1

OVGP1 is a mucin belonging to a protein family named glycoside hydrolase 18, which shares a chitinase catalytic domain that is not active in OVGP1 [182,196], due to the lack of an essential glutamic acid residue in the N-terminal domain of the protein [119,197]. The protein contains a mucin-type tandem repeat, a signal peptide and several post-translational modification sites involved in secretion [119], as well as a clatrhin box associated with endocytosis. In human, baboon, porcine and bonnet a Class III PDZ-binding domain has been reported, which suggests that OVGP1 could be part of a multi-protein complex [198].

Analysis of the amino acid sequence of OVGP1 exhibited that the N-terminal region of mature OVGP1 shares a high degree of identity (77–84%) and similarity (86–90%) with other species. In contrast, the C-terminal region has a low degree of identity (37–63%) and similarity (50–75%), as well as several insertions/deletions in its sequence [113]. A comparative analysis of the alignment of deduced aminoacidic sequences of several mammalian OVGP1 proteins revealed the existence of five differentiated regions (A–E). Region A, which corresponds to the N-terminus, has a high degree of identity in monotremes, marsupials and placental mammals. Region B shows low identity among different mammals and contains multiple insertions/deletions. Region C is an insertion present only in the mouse, and region E is typical of human, chimpanzee and orang-utan [3]. Analysis, at both the molecular and physiological level, of the role of the C-terminal region of OVGP1 in fertilization provided a model in which OVGP1 binds to ZP via its highly conserved A region, which may also provide an anchor for additional oviduct proteins. The C-terminal regions of OVGP1 modulate its binding to the ZP, regulate OVGP1 activity and account for the reproductive role of OVGP1 in different mammalian orders [113].

The apparent molecular mass of OVGP1 glycoprotein on reducing SDS-PAGE ranges from 66 to 350 kDa in different species [177]. Carbohydrates make a large contribution to the weight of the protein. At least two isoforms of the protein [119], as well as polymorphism in the gene sequence [3,186,187], have also been reported. Oddly, a 95 kDa bovine OVGP1 isoform has been identified inside EVs, which is more than the 75 kDa found in the OF [68]. 

### 4.3. Effect of OVGP1 Localized in Eggs and Embryos

OVGP1 has been associated with the ZP of embryos and oocytes from different species, both in homologous and heterologous systems, as well as with blastomere plasma membranes, inside blastomeres, endosomes, lysosomes, multivesicular bodies and with the perivitelline space, as shown in Table 4, suggesting the protein role in the regulation of fertilization and early embryo development. The association of OVGP1 to the ZP is stable and uniform and is maintained until day 7 in bovine uterine embryos [199] and was also detected in 7–day old hatched porcine uterine embryos [127]. The protein complex formed by OVGP1 and heparin-like glycosaminoglycans is an important regulator of OVGP1 binding to the ZP since their union is considered reversible [200]. Recombinant porcine OVGP1 was detected through the whole thickness of the ZP of porcine and bovine oocytes when it was present during the IVF [113,201]. It was also found bound to the ZP in 9-day old bovine embryos, but only if the in vitro culture medium contained OVGP1 until day 3.5 post-insemination [201]. Using OF during the incubation of in vitro-produced bovine embryos, OVGP1 was localized in the perivitelline space and in blastomeres of both 4–6 cell and morula-stage embryos, but not bound to the ZP [98]. This agrees with the previously described reversible nature of OVGP1-ZP binding [200], whereby only two bovine OVGP1 proteins of 75 and 95 kDa were reported to bind porcine ZP [200]. In mouse, OVGP1 was associated with the perivitelline space of oocytes and embryos [130,202,203] but only a peanut agglutinin-binding glycoform of the protein was associated with the ZP [203].

As regards the role of OVGP1 in fertilization, ZP exposure to OVGP1 generally results in modification of the ZP and an increase in resistance to digestion by proteolytic enzymes (enzymatic hardening of the ZP) [149,167,210,211,212,213,214,215,216,217], which, in turn, contributes to the control of polyspermy [200] and improves the efficiency of in vitro fertilization [113,218]. Introducing specific antibodies against the protein blocked the observed biological effect [146,167]. Although one publication reported no ZP hardening after the treatment of porcine oocytes with OVGP1 protein purified from pig OF [167], the ZP of porcine oocytes exposed to both, purified recombinant porcine and rabbit OVGP1 increased ZP resistance to enzymatic proteolysis in a dose-dependent manner [113] but only homologous recombinant protein increased the efficiency of fertilization. Thus, the correlation between the induction of hardening and improved IVF rates only occurs in homologous systems. Indeed, human OVGP1 enhances sperm binding to the ZP, whereas heterologous OVGP1 (baboon) inhibits this effect even though the two proteins are 94% identical [137] the same being observed in hamster [219]. The presence or absence of specific regions in the C-terminus of OVGP1 affects its association with the ZP, as well as its ability to remodel the matrix and so its effect on fertilization [113]. The pre-treatment of oocytes with OVGP1 increases sperm-egg binding [207] and zona penetration rates in hamster [220] and human [137], but the opposite effect occurs in pig [200].

Moreover, the presence of the glycoprotein inside the embryo suggests it plays role in embryo development. Notably, antibodies against a C-terminal peptide of OVGP1 inhibit early mouse development, so that embryos do not progress from the 2-cell stage [221]. Indeed, purified porcine OVGP1 enhanced cleavage and the blastocyst formation rate in in vitro-produced porcine embryos when they were cultured in a medium supplemented with the protein for 48 or 144 h [218]. Using purified porcine OVGP1 during preincubation and IVF increased their post-cleavage development to blastocyst [167]. Similarly, the supplementation of the IVF medium with an OVGP1-enriched fraction of OF increased cleavage rates in ovine [222]. By contrast, using ovine OVGP1 in IVC decreased the proportion of zygotes undergoing the first cleavage, increased the time needed for blastocyst formation, and also the mean number of nuclei per blastocyst, resulting in blastocysts developing more similarly to the those produced in vivo [222,223]. Moreover, supplementing the IVM, IVF and IVC medium with purified OVGP1 from goat oviductal tissue increased the cleavage rate, and morula and blastocyst yield at the lowest used concentration (10 µg/mL), but had an inhibitory effect at higher concentrations (50 and 100 µg/mL) [149]. A feline recombinant OVGP1 expressed in a bacterial expression system did not affect cleavage, morula or blastocyst rates in cat, but increased the relative mRNA level of the GJA1 gen, an embryo quality marker [141]. In addition, the use of recombinant porcine OVGP1 during bovine IVF or embryo IVC did not affect cleavage or blastocyst yield but resulted in embryos with an increased relative abundance of mRNA of embryo quality marker genes as: aquaporins (AQP3), transcription factor (ATF4), cell adhesion proteins (DSC2) and methyltransferase (DNMT3A) [201].

The reported effects of OVGP1 glycoprotein differs from species to species. It is also important to bear in mind the great variability in the experimental design used: different OVGP1 sources, protein concentrations, times of OVGP1 exposure, among others. Furthermore, the recent discovery of OVGP1 in EVs could determine the protein activity controlled by this system, which has not been described to date. In addition to the species-specific effect, all the above could explain the varied effects described for this oviductal glycoprotein. New gene editing techniques used in species other than murine, might help clarify in determining role of this protein in different species of mammals during periconception period. 

## Figures and Tables

**Table 1:** Chronology of embryo development in different species.

	HUMAN	MOUSE	PIG	COW	SHEEP	MARE	CAT	BITCH	GUINEA PIG	RABBIT
2 cells	1 (in vitro) [28]	1 [29]	14–16 h [30]	24–30 h [31]	1.5 [32]	20 h [33]	2.5 [34]	4.5–5.5 [35]	2 [36]	16–17 h [37]
8 cells	3 (in vitro) [38]	2.5 [29]	2 [30]	3.5 [39]	2 [32]	3 [40]	3 [34]	4.5–5.5 [35]	4 [36]	29–30 h [37]
Morula	4 (in vitro) [28]	3 [29]	3.5 [30]	5–6 [31]	4 [32]	4 [40]	5 [34]	10 [41]	5 [36]	2.5 [42,43]
Blastocyst	5 (in vitro) [28]	3.5 [29]	5 [30]	7 [31]	6 [44]	6 [40]	6–9 [34]	12–13 [41]	6 [36]	3 [43]
Hatching	6–7 (in vitro) [45]	4.5 [46]	6 [30]	8 [31]	8 [44]	7–8 [40]	12 [47]	19–20 [48]	6 [49]	4 (in vitro) [37] no hatching until implantation in vivo [50]
Conceptus reaches the uterus	3.5 [51]	3 [29]	2 [30]	4 [31]	4 [44]	5.5–6.5 [40,52]	5–6 [34]	7–10 [41,53,54]	4.5 [36]	3.5 [42]
Conceptus reaches the uterus (stage)	Morula [51]	Morula [29,55]	4–8 cells [30]	16 cells [31]	Morula (16–32 cells) [44]	Blastocyst [40]	Compact morula or blastocyst [34]	Compact morula or blastocyst [56]	8–16 cells [36,57]	Blastocyst [42]
Implantation	6–10 [58]	4.5 [55]	14–18 [59]	19 [31]	16 [44]	~40 [59]	13–14 [60]	From 17–18 [61]	6–7 [49]	7.5 [42]

Time is indicated in days unless otherwise indicated.

**Table 2:** Common protein-ligand interactions in different species.

OF Ligands		Receptor in Blastomeres		Citations
Annexin A1	ANXA1	Epidermal growth factor receptor	EGFR	[98]
Apolipoprotein C-III	APOC3	Syndecan 2	SDC2	
Complement component 3	C3	CD19 molecule	CD19	[99,100]
		CD81 molecule	CD81	
Calreticulin	CALR	Integrin, alpha V	ITGAV	
Fibrinogen gamma chain	FGC	Integrin, alpha V	ITGAV	[101]
		Integrin, beta 1 (fibronectin receptor, beta polypeptide)	ITGB1	
Heat shock protein 90 kDa alpha (cytosolic), class A member 1	GPI	Cystic fibrosis transmembrane conductance regulator	CFTR	
		Epidermal growth factor receptor	EGFR	
Lactotransferrin	LTF	Transferrin receptor	TFRC	[102]
Glucose-6-phosphate isomerase	GPI	Autocrine motility factor receptor	AMFR	

**Table 3:** Mammalian species where OVGP1 has been detected.

Species	Reference
Baboon *(Papio anubis)*	[134,135,136,137,138]
Cat *(Felis catus)*	[139,140,141]
Chimpanzee *(Pan troglodytes)*	[142]
Cow *(Bos taurus)*	[7,125,143,144,145,146]
Dog *(Canis lupus familiaris)*	[147]
Goat *(Capra ibex)*	[148,149]
Hamster *(Mesocricetus auratus)*	[118,124,150,151,152,153,154,155,156]
Human *(Homo sapiens)*	[129,131,157,158,159]
Macaque *(Macaca mulatta)*	[160,161]
Mouse *(Mus musculus)*	[120,162,163]
Pig *(Sus scrofa)*	[164,165,166,167,168]
Rabbit *(Oryctolagus cuniculus)*	[169,170,171]
Sheep *(Ovis aries*)	[172,173,174,175,176]

**Table 4:** **OVGP1 protein detected bonded to the zona pellucida (ZP), oocytes and embryos of different mammalian species.** OVGP1 comes from oviductal oocytes (in vivo) or ovarian oocytes exposed to OF, purified protein from oviductal tissue explant or recombinant protein (in vitro).

	OVGP1 Source	In Vivo/In Vitro	Technique	ZP	Oocyte	Embryo	Reference
Baboon *(Papio anubis)*	Baboon	in vivo/in vitro	OM	+	+ (PVS)	2–4 cells. + (ZP, PVS)	[137,156,204]
			EM	+	+ (PVS, PM, O)	+ (ZP, PVS, BO)	
	Human	in vitro	OM	+	/	/	[137]
Human *(Homo sapiens)*	Human	in vitro	OM	+	/	/	[137]
	Baboon	in vitro (Hemizonae)	OM	+	/	/	[137]
Hamster *(Mesocricetus auratus)*	Human	in vitro	OM	+	+ (PVS)	/	[205]
	Hamster	in vivo/in vitro	OM	+	-	/	[96,124,150,151,154,156,205,206,207]
			EM	+	+ (PVS, PM, OV)	2–8 cells. + (ZP, E, L, MVB)	
			WB	+	/	/	
Pig *(Sus scrofa)*	Pig	in vivo/in vitro	OM	+	+ (ZP, PM, O)	/	[113,127,200]
			EM	+	+ (PVS, PM, MVB)	Day 2–7 + (ZP, PVS, PM, BM)	
			MS	+	/	/	
	Cow	in vitro	OM	+	/	/	[208]
Cow *(Bos taurus)*	Cow	in vivo/in vitro	OM	+	-	Day 7 + (ZP) Day 4–6 and morula + (PVS, BC)	[98,199,209]
			MS	/	/	+	
	Pig	in vitro	OM	+	+ (ZP)	+ (ZP, PVS, BM, BC)	[201]
Sheep *(Ovis aries)*	Sheep	in vivo/in vitro	OM/EM/WB	+	+ (PVS)	+ (ZP, PVS, BC)	[173,174]
Mouse *(Mus musculus)*	Mouse	in vivo/in vitro	OM	+/−	+ (PVS)	2 cells + (ZP, PVS)	[130,162,202,203]
			EM	−	+ (PVS)	/	
			WB	+/−	+/−	/	

OM: optical microscopy, EM: electron microscopy, WB: Western-blot, MS: Mass spectrometry, ZP: zona pellucida, PVS: perivitelline space, PM: plasma membrane, O: ooplasm, OV ooplasm vesicles, BM: blastomere membrane, B: blastomere cytoplasm, E: endosome, L: secondary lysosome, MVB: multivesicular bodies, (+): positive labelled, (−): negative labelled.

## Supplementary Materials

The following are available online at https://www.mdpi.com/2218-273X/10/12/1690/s1, Table S1: Deletes peptides *Sus scrofa*, Table S2: DEGs oviduct cow + mare + so, Table S3: Exosomal vs secreted, Table S4: Conserved interactions, Table S5: Human interactions, Table S6: Cow interactions, Table S7: Pig interactions.

## Date Availability

All data generated or analyzed during this study are included in this published article (and its supplementary information files).
